# Maximum oxygenation during surgical abortion with sedatives and analgesics – a simple suggestion from an anesthesiological perspective

**DOI:** 10.1186/s40981-025-00775-y

**Published:** 2025-02-15

**Authors:** Keisuke Yoshida, Riho Yazawa, Satoki Inoue

**Affiliations:** 1 Department of Anesthesiology, Fukushima Medical University, 1, Hikariga-Oka, Fukushima City, Fukushima, 960-1297 Japan; 2 Department of Obstetrics and Gynecology, Ohtanishinouchi Hospital, Koriyama, Japan

**Keywords:** Surgical abortion, Anesthesia, Sedation, Patient safety

*To the editor,*

The World Health Organization guidelines for abortion care recommend vacuum aspiration as the preferred method for surgical abortion, performed with paracervical nerve blocks to avoid general anesthesia [1]. However, in Japan, dilation and curettage (D&C) remains a common abortion procedure, and obstetricians often opt for general anesthesia. Consequently, general anesthetic agents (e.g., sedatives such as propofol, midazolam, thiopental, and dexmedetomidine, as well as analgesics such as fentanyl and pentazocine) are often used by obstetricians. The level of sedation/anesthesia ranges from minimal sedation to general anesthesia, and variations in drug dosage can lead to serious airway and respiratory complications. Therefore, general anesthesia or sedation should not be used lightly. If avoidance is not possible, surgical abortion with sedation/analgesia should ideally be performed in a properly monitored environment under the supervision of an anesthesiologist or emergency physician. This practice is known as monitored anesthesia care [2].

However, it is difficult to immediately create an ideal environment in all facilities where surgical abortions are performed, and obstetricians may have no choice but to use general anesthetic agents by themselves during these procedures. Therefore, from an anesthesiologist’s perspective, we propose that maximum oxygenation be added to the standard protocol used in each facility for surgical abortion with sedatives/analgesics. Specifically, at least 3 min before the administration of the drugs [3], oxygen at the highest possible concentration and flow rate should be administered through a mask with a reservoir bag or a Jackson-Rees circuit placed in close contact with the face. This approach can mitigate the risk of accidental hypoxia, which is a significant cause of cardiac arrest in young healthy adults, especially in those with no prior cardiac history. Additionally, since it does not require any special skills, it can be immediately implemented in any facility. It is the same concept as preoxygenation, which is routinely performed prior to induction of general anesthesia to address apnea. It has been shown that healthy adults can tolerate apnea/non-ventilation for approximately 8 min without developing hypoxemia if adequately preoxygenated [3]. Obstetricians, not only anesthesiologists, should note that the time to hypoxemia is shortened by more than half in obese patients and in pregnant women because of reduced functional residual capacity [3, 4]; however, surgical abortion is typically performed in early pregnancy, so they can be considered to be almost normal adults.

Of note, maintaining oxygenation does not guarantee normal respiratory status; thus, maximum oxygenation alone cannot prevent airway or respiratory complications. Therefore, strict monitoring of respiratory status and timely interventions such as respiratory support are necessary, even after adopting this approach. However, we believe that, for surgical abortions completed in a relatively short time, proper preoxygenation can provide additional time before the onset of hypoxemia, which serves as an important safety measure when sedation/anesthesia is administered by non-anesthesiologists or emergency physicians.
